# Lack of association between polymorphisms of the *IL18R1 *and *IL18RAP *genes and cardiovascular risk: the MORGAM Project

**DOI:** 10.1186/1471-2350-10-44

**Published:** 2009-05-27

**Authors:** Marie-Lise Grisoni, Carole Proust, Mervi Alanne, Maylis DeSuremain, Veikko Salomaa, Kari Kuulasmaa, François Cambien, Viviane Nicaud, Per-Gunnar Wiklund, Jarmo Virtamo, Frank Kee, Laurence Tiret, Alun Evans, David-Alexandre Tregouet

**Affiliations:** 1 INSERM, UMR_S 937, F-75013, Paris, France; 2 UPMC Univ Paris 06, UMR_S 937, F-75013, Paris, France; 3 Department of Molecular Medicine, National Public Health Institute, Helsinki, Finland; 4 Department of Health Promotion and Chronic Disease Prevention, National Public Health Institute, Helsinki, Finland; 5 Department of Internal Medicine, University of Umeå, Umeå, Sweden; 6 Centre Department of Epidemiology and Public Health, Queen's University of Belfast, Belfast, UK

## Abstract

**Background:**

Interleukin-18 is a pro-inflammatory cytokine suspected to be associated with atherosclerosis and its complications. We had previously shown that one single nucleotide polymorphism (SNP) of the *IL18 *gene was associated with cardiovascular disease (CVD) through an interaction with smoking. As a further step for elucidating the contribution of the IL-18 pathway to the etiology of CVD, we here investigated the association between the genetic variability of two IL-18 receptor genes, *IL18R1 *and *IL18RAP*, with the risk of developing CVD.

**Methods:**

Eleven tagging SNPs, 5 in *IL18R1 *and 6 in *IL18RAP*, characterizing the haplotypic variability of the corresponding genes; were genotyped in 5 European prospective CVD cohorts including 1416 cases and 1772 non-cases, as part of the MORGAM project. Both single-locus and haplotypes analyses were carried out to investigate the association of these SNPs with CVD.

**Results:**

We did not find any significant differences in allele, genotype and haplotype frequencies between cases and non-cases for either of the two genes. Moreover, the search for interactions between SNPs located in different genes, including 5 *IL18 *SNPs previously studied in the MORGAM project, and between SNPs and environmental factors remained unfruitful.

**Conclusion:**

Our analysis suggests that the variability of *IL18R1 *and *IL18RAP *genes are unlikely to contribute to modulate the risk of CVD.

## Background

Interleukin-18 (IL-18) is a pro-inflammatory molecule that has been shown to be involved in the susceptibility of several human complex diseases such as immune diseases, type I diabetes and cardiovascular diseases (CVD) [1]. The hypothesized mechanism by which IL-18 may be linked to CVD risk is related to atherosclerosis and its complication [2-9]. Consistent with this hypothesis, highest IL-18 levels were shown to be associated with increased carotid intima-media thickness [10] and with cardiovascular mortality in a cohort of patients with coronary artery disease [7,8]. As initiating the IL-18 signalling cascade requires the formation of a heterodimeric receptor (IL-18R) composed of a binding chain α, termed IL-18R1 or IL-18Rα, and a signal transducing β chain, termed IL-18RAP (for IL-18 receptor accessory protein) or IL-18Rβ [11-13], both IL-18R1 and IL-18RAP might also be good candidates for CVD. IL-18R1 and IL18-RAP are expressed on a variety of cells including macrophages, T lymphocytes, and natural killer cells, which are ascribed a key role in atherosclerotic plaque rupture [14,15]. The hypothesized relationship between IL-18 receptors and CVD could be reinforced by showing that single nucleotide polymorphisms (SNPs) within the *IL-18R1 *and *IL18-RAP *genes could be associated with CVD.

While *IL18R1 *and *IL18RAP *polymorphisms have been found associated with diseases such as schizophrenia, HSV1 seropositivity and atopic asthma [16,17], little is known about their contribution to CVD. *IL18R1 *and *IL18RAP *tag SNPs have been investigated in a German cohort of coronary artery disease patients in relation to cardiovascular mortality [9] but no association was observed. However, the number of patients experiencing the end point of interest was moderate (n = 142) and could have limited the power to detect mild genetic effects.

Therefore, in order to get a better insight of the contribution of *IL18R1 *and *IL18RAP *genes on CVD risk, we investigated the association of tagging SNPs within these genes with the risk of CVD in the MORGAM Project [18], a collaborative study pooling several European (Finns, Swedish, Northern Irish and French) prospective population cohorts expected to provide ample power to detect moderate genetic effects. In addition, in light of our recent finding suggesting that smoking could modulate the effect of *IL18 *SNPs on the risk of CVD [19], we were interested in testing whether *IL18R1 *and *IL18RAP *SNPs could also interact with smoking, an hypothesis that has never been investigated before.

## Methods

### MORGAM study populations

MORGAM is a multinational collaborative project of several European population cohorts, which were followed up for cardiovascular disease and whose descriptions have already been published [18]. The present report was based on the analysis of five cohorts, two from Finland (FINRISK, ATBC), one from France and one from Northern Ireland, (both issued from the PRIME Study), and one from Sweden. The FINRISK cohort comprised two surveys with baseline investigations five years apart (1992 and 1997). Both were pooled in this report after having checked for consistency across surveys and the analysis was adjusted for survey. All individuals were followed up over a median period of 6.0 (maximum 10.9) years for mortality and for several cardiovascular outcomes including thromboembolic, coronary heart disease (CHD) and stroke events. To facilitate the study of multiple endpoints and to reduce genotyping costs, a case-cohort design [20,21] has been adopted in MORGAM. In each population cohort, a subset of individuals from the whole cohort was randomly selected independently of disease status to be part of a subcohort according to population-specific sampling probabilities. These probabilities were dependent on sex and age such that older subjects had a higher selection probability and that age distributions were similar in cases and in the subcohort. All individuals were followed up for clinical outcome that was obtained mostly from national death register, MONICA and hospital discharge registers, and regional health information system [18]. Genotyping was however restricted to all subcohort members and to all additional subjects who were not part of the subcohorts but who experienced cardiovascular outcomes during the follow-up. Subjects with cardiovascular events prior to the baseline examination were excluded from the analysis.

Globally, the subcohort was composed of 2016 subjects. In this study, we were interested in stroke events (ischemic and hemorrhagic) and CHD events (including definite and possible acute myocardial infarction, coronary death, unstable angina pectoris and cardiac revascularization). Both ischaemic and hemorrhagic stroke were included in the analysis because there is often insufficient data to separate between the subtypes in this population-based study. About 80% of the strokes are ischaemic in these populations. Detailed events definition is available in the cohort descriptions [22]. Both fatal and non-fatal events were considered and, when multiple events occurred during the following-up, only the first one was studied. The case-cohort data set was composed of 1416 cases (244 subjects in the subcohort and 1172 subjects from outside the subcohort) and 1772 non-cases.

### Quality control of the extracted DNA

A central core laboratory aliquotted the DNA samples from all cohorts except the two cohorts of the prospective study PRIME. Fluorescent label PicoGreen (Invitrogen, Carlsbad, CA, USA) was used to normalize the DNA concentration prior to aliquotting. The samples were genotyped with quality control marker panel including sex-specific markers (one marker in the Y-chromosome, 2 in X-chromosome and 3 autosomal markers). Genotype-inferred gender was compared with the clinical records. The presence of discrepancies may indicate either that sample labels have been mixed or contamination with another DNA. This can be observed only when a sample of men has been mixed or swapped with a sample of women. Therefore, genotyped samples also included ~2% known duplicates, 5% blinded duplicates and negative controls. We then excluded all discrepant samples. The genotyping success rate was at least 98% for all control markers and we observed less than 0.25% genotyping errors based on the duplicate comparison. This quality control process was performed only once and independently of any planned SNP-specific genotyping.

### Genotyping

SNPs genotyped in the present study were exactly those that were previously studied in the AtheroGene cohort [9] where detailed description of the selection criteria can be found. Briefly, these were haplotype tagging SNPs selected to capture more than 95% of the haplotype diversity of the regulatory, coding and flanking intronic regions of the *IL18R1 *and *IL18RAP *genes according to the information available in the Innate Immunity Programs for Genomic Applications database . These included 5 SNPs in *IL18R1*, rs1420098 (T-6/in1C), rs1420096 (T-17/in8C), rs11465656 (+43Ins/Del), rs3732127 (G+404C), rs11465660 (C+968A), and 6 SNPs in *IL18RAP*, rs11465670 (T-1301C), rs4851581 (A-992G), rs1420106 (G-697A), rs1420105 (C-622T), rs11465673 (T-366C), rs11465702 (A+55/inG). In addition to these 11 SNPs, 5 *IL18 *SNPs previously studied in the MORGAM project [19] were also investigated in the search for interaction among SNPs of the IL-18 pathway. These were rs1946519 (G-887T), rs360717 (C-105T), rs549908 (S35S (A/C)), rs5744292 (A+183G) and rs4937100 (T+533C). Genotyping was performed using the TaqMan 5' nuclease detection assay on a 7000 Sequence Detection System (Applied Biosystems). All information for genotyping (PCR primers, probes, conditions of amplification and hybridization) can be found on the GeneCanvas website .

### Statistical analysis

Deviation from Hardy-Weinberg equilibrium (HWE) was tested by a standard χ^2 ^with 1 degree of freedom and allele frequencies were estimated using the gene-counting method in each subcohort separately. Association of *IL18R1 *and *IL18RAP *SNPs with the case/non-case status was tested using a logistic regression model including the inverse of the sampling probability as an offset [21]. In most analyses, additive allele effects were assumed except for rare alleles where dominant models were considered. In order to deal with linkage disequilibrium (LD) between SNPs of the same gene, haplotype analyses were further carried out using the THESIAS software [23]. Tests of association between haplotypes and the case/non-case status were performed using the likelihood ratio test.

Analyses were performed separately in each population and were adjusted for age at baseline, baseline status, gender and smoking status when appropriate. Homogeneity of the associations across cohorts was assessed by use of the Mantel-Haenszel statistics [24,25] that was subsequently used for estimating pool odds ratio (OR) in the whole study. The Mantel-Haenszel method is a standard way to estimate averaged OR by weighting each log(OR) obtained in a given population by the inverse of their variance.

For each gene, adjustment for multiple testing was made by applying a Bonferroni correction for the number of inferred haplotypes times the number of population cohorts.

The DICE method [26,27] was used to investigate SNP × SNP interactions as well as interaction of SNPs with covariates including age, gender, smoking status and body mass index. DICE is an algorithm that explores in an automated way all combinations of one, two or three covariates, that could be SNP or environmental factors, acting either additively or in an interactive way. The selection for the most parsimonious model of interaction, if any, is based on a Information Criterion. This search was first carried out separately in each cohort and then in the combined sample while adjusting for cohorts.

## Results

A brief description of the five populations used for this analysis is given in Table 1, with a total number of 1772 non-cases and 1416 cases of CVD. Three cohorts, ATBC, PRIME/France and PRIME/N.Ireland included men only. Among the 1,416 cases, 923 experienced only a CHD event, 425 only a stroke event and 68 experienced both events. The first event was fatal for 258 CHD cases and 55 stroke cases.

**Table 1:** Description of the MORGAM cohorts

	Number of individuals									
		
Cohort	Subcohort^a^	Incident cases outside subcohort	All	Non-cases	CHD^b^	Stroke^b^	Smokers^c^	Males	Age at baseline^d ^(years)	Follow-up median/max (years)
FINRISK (Finland)	700 (609/91)	476	1176	609 (51.8%)	382 (32.5%)	185 (15.7%)	285 (24.2%)	827 (70.3%)	57.0 [25–74]	8.07/10.96
ATBC (Finland)	849 (718/131)	421	1270	718 (56.5%)	343 (27.0%)	209 (16.5%)	1007 (79.3%)	1270 (100%)	63.4 [54–76]	7.12/8.29
Sweden	136 (128/8)	59	195	128 (65.6%)	29 (14.9%)	38 (19.5%)	37 (19.0%)	121 (62.0%)	55.8 [24–73]	6.04/10.87
PRIME N.Ireland	143 (135/8)	108	251	135 (53.8%)	103 (41.0%)	13 (5.2%)	66 (26.3%)	251 (100%)	54.9 [50–60]	5.14/5.99
PRIME France	188 (182/6)	108	296	182 (61.5%)	98 (33.1%)	16 (5.4%)	73 (24.7%)	296 (100%)	55.3 [49–60]	5.28/7.61
Entire	2016 (1772/244)	1172	3188	1772 (55.6%)	955 (30.0%)	461 (14.5%)	1468 (46.0%)	2765 (86.7%)	59.2 [24–76]	6.33/10.96

^a ^Total number of individuals in the random subcohort (non-cases/cases)

^b ^In presence of several events, only the first event was considered.

^c ^Daily cigarette smoker at baseline

^d ^Mean age [min - max]

All analyses were performed separately in each cohort but, for ease of presentation, most of the results presented are those obtained in the whole study, after having checked the homogeneity of the associations.

### *IL18R1 *gene

**Figure 1:**
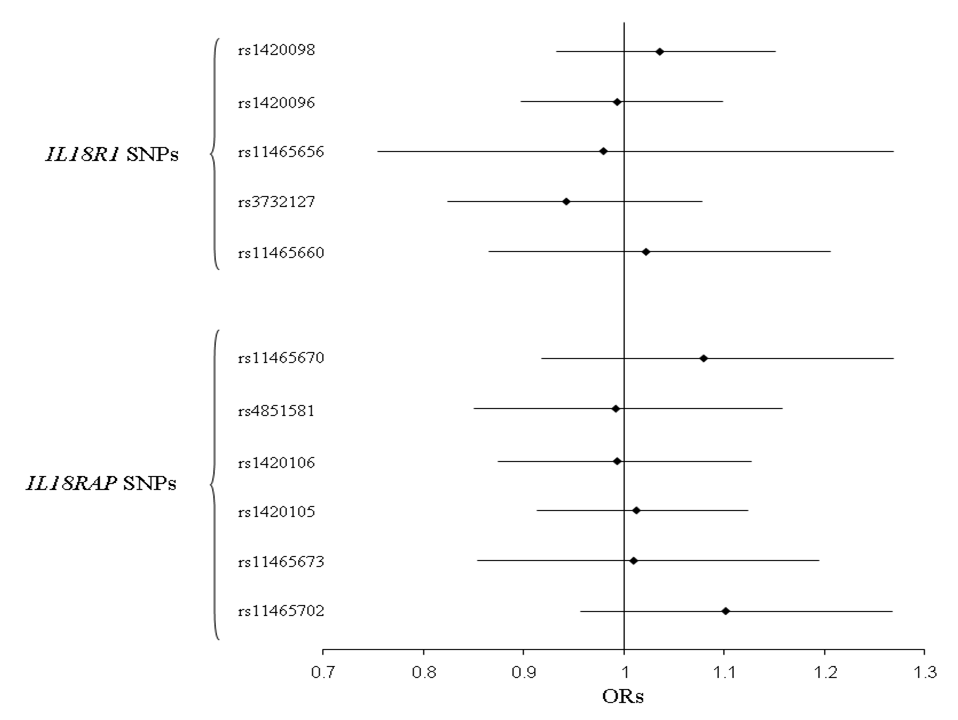
**Association of *IL18R1 *and *IL18RAP *SNPs with CVD risk in the whole MORGAM cohorts**. Combined ORs [95% confidence interval] were obtained by use of the Mantel-Haenszel method and adjusted for age, gender and smoking status.

In each MORGAM subcohort, all genotype distributions were compatible with Hardy Weinberg equilibrium (HWE). For three SNPs, allele frequencies differed across subcohorts (Additional File 1). The SNP showing the strongest difference (p < 0.0001) across subcohorts was the rs11465660, the frequency of the rs11465660-A allele varying from 0.07 in France to 0.15 in Sweden. None of the *IL18R1 *SNPs was consistently associated with CVD (Table 2 – Figure 1). The rs3732127-C allele was slightly more frequent in Sweden cases than in Sweden non-cases (0.22 vs 0.14), but this association (p = 0.037), not found in others cohorts, was no longer significant after correction for multiple testing

**Table 2:** Allele frequencies^a ^of the *IL18R1 *and *IL18RAP *SNPs in the MORGAM cohorts

		FINRISK N = 1176		ATBC N = 1270		Sweden N = 195		PRIME/N.Ireland N = 251		PRIME/France N = 296	
Gene	SNP	Non-cases N = 609	Cases^b^ N = 567	Non-cases N = 718	Cases N = 552	Non-cases N = 128	Cases N = 67	Non-cases N = 135	Cases N = 116	Non-cases N = 182	Cases N = 114

*IL18R1*	rs1420098 (T/C)	0.37	0.38	0.39	0.41	0.43	0.41	0.42	0.42	0.41	0.37
	rs1420096 (T/C)	0.46	0.47	0.46	0.47	0.52	0.43	0.50	0.48	0.55	0.53
	rs11465656 (I/D)	0.04	0.04	0.04	0.04	0.03	0.02	0.04	0.06	0.05	0.03
	rs3732127 (G/C)	0.21	0.18	0.17	0.17	0.14	0.22*	0.17	0.19	0.19	0.16
	rs11465660 C/A)	0.10	0.10	0.12	0.12	0.13	0.13	0.11	0.07	0.07	0.07

*IL18RAP*	rs11465670 (T/C)	0.09	0.09	0.11	0.12	0.11	0.16	0.13	0.15	0.13	0.13
	rs4851581 (A/G)	0.14	0.15	0.15	0.13	0.08	0.03	0.08	0.10	0.09	0.11
	rs1420106 (G/A)	0.18	0.20	0.20	0.19	0.21	0.19	0.20	0.18	0.22	0.28
	rs1420105 (C/T)	0.46	0.46	0.46	0.47	0.51	0.43	0.50	0.48	0.53	0.53
	rs11465673 (T/C)	0.09	0.10	0.12	0.12	0.13	0.14	0.11	0.07	0.07	0.06
	rs11465702 (A/G)	0.19	0.16*	0.15	0.14	0.12	0.16	0.14	0.14	0.13	0.11

^a ^Allele frequency of the minor allele.

^b ^Cases: CHD and strokes were combined

*: Significant cases/non-cases comparison at p < 0.05

**Figure 2:**
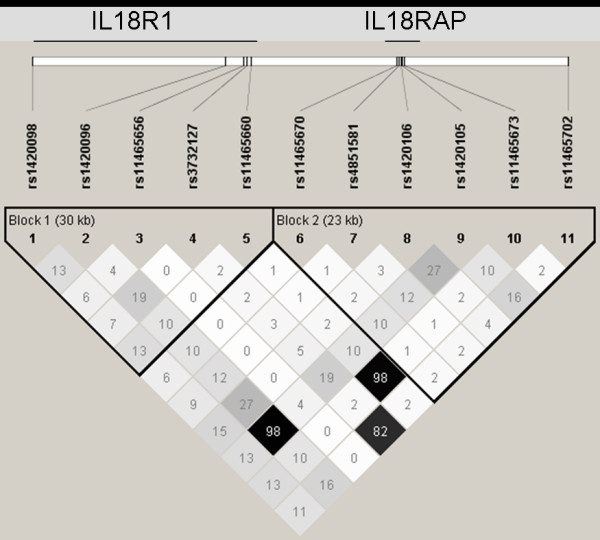
**Pairwise Linkage Disequilibrium between the *IL18R1 *and *IL18RAP *SNPs**. Pairwise Linkage Disequilibrium (LD) expressed in terms of r^2 ^and estimated in the whole MORGAM cohort using the Haploview software (Barrett JC, Fry B, Maller J, Daly MJ. Haploview: analysis and visualization of LD and haplotype maps. Bioinformatics 2005; 21:263–265). The pattern of LD was homogeneous across the five European cohorts (data not shown).

**Figure 3:**
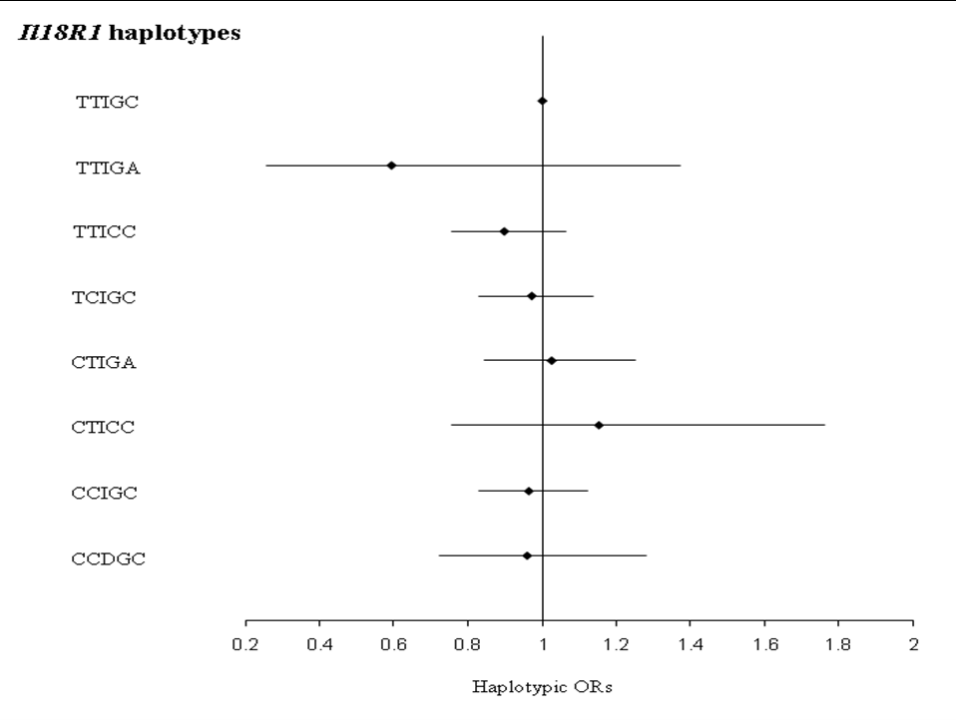
**Association between *IL18R1 *haplotypes and CVD risk in the whole MORGAM cohorts**. Haplotypic ORs [95% confidence interval] adjusted for cohorts, age, gender and smoking habits are shown by comparison to the reference TTIGC haplotype. The global test of association was not significant (χ^2 ^= 7.231 with 7 df, p = 0.405). Polymorphisms are ordered according to their position on the genomic sequence.

LD pattern between *IL18R1 *SNP is shown in Figure 2. Eight haplotypes with frequency greater than 0.02 in at least one subcohort were inferred from the 5 *IL18R1 *tag SNPs (Table 3). The haplotypic structures were broadly homogenous across the five cohorts. The rs11465656-Del allele was the only allele carried by only one haplotype, homogeneously across subcohorts. No association between *IL18R1 *haplotypes and CVD risk was observed in any of the MORGAM cohorts, with the p-value of the global test of association ranging from 0.138 to 0.877 in FINRISK and ATBC, respectively (Additional File 2 – Tables 1 &2). In the whole MORGAM cohort, no specific haplotype was associated with the disease status (Figure 3).

**Table 3:** *IL18R1 *gene haplotype frequencies in the MORGAM subcohorts

Polymorphisms									
					
rs1420098	rs1420096	rs11465656	rs3732127	rs11465660	FINRISK N = 700	ATBC N = 849	Sweden N = 136	PRIME/N.Ireland N = 143	PRIME/France N = 188
T	T	I	G	C	0.239	0.256	0.200	0.220	0.200
T	T	I	G	A	0.009	0.005	0.027	0.016	0.028
T	T	I	C	C	0.195	0.151	0.127	0.145	0.143
T	C	I	G	C	0.186	0.196	0.220	0.198	0.218
C	T	I	G	A	0.086	0.115	0.106	0.083	0.037
C	T	I	C	C	0.010	0.018	0.018	0.036	0.034
C	C	I	G	C	0.232	0.211	0.263	0.251	0.262
C	C	D	G	C	0.037	0.041	0.028	0.051	0.050

### *IL18RAP *gene

Again, all genotypic distributions were compatible with HWE in all MORGAM subcohorts. All *IL18RAP *SNPs exhibited differences in allele frequencies across subcohorts (Additional File 1). In particular, the rs4851581-G allele was almost twice as common in Finnish populations when compared to other cohorts. None of the *IL18RAP *SNPs was consistently associated with CVD (Table 2 – Figure 1). In the FINRISK cohort, the rs11465702-G allele was slightly less frequent in cases than in non-cases (0.16 vs 0.19, p = 0.048), but this association was not observed in others cohorts and did not remain significant after multiple testing correction

**Figure 4:**
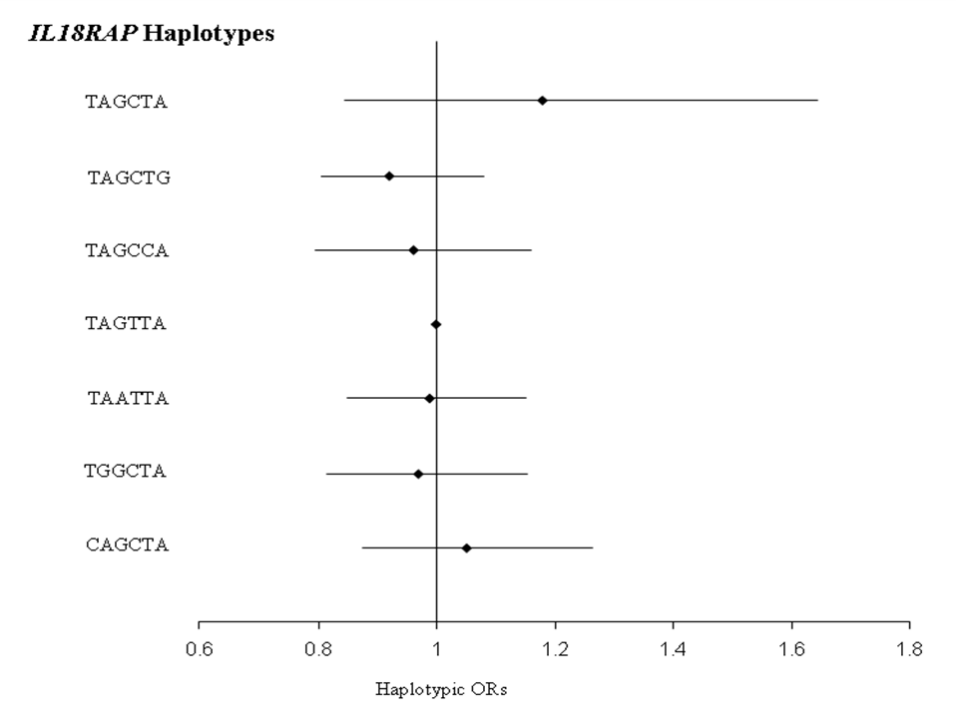
**Association between *IL18RAP *haplotypes and CVD risk in the whole MORGAM cohorts**. Haplotypic ORs [95% confidence interval] adjusted for cohorts, age, gender and smoking habits are shown by comparison to the reference TAGTTA haplotype. The global test of association was not significant (χ^2 ^= 1.763 with 6 df, p = 0.940). Polymorphisms are ordered according to their position on the genomic sequence.

The 6 *IL18RAP *SNPs generated 7 haplotypes with frequency higher than 1%. These common haplotypes were observed in all subcohorts but with different frequencies (Table 4). Except for the rs1420105, all rare alleles were carried by only one haplotype, homogenously across cohorts. No association between *IL18RAP *haplotypes and CVD risk was detected in any cohort (p-values ranging from 0.154 to 0.848 in Sweden and ATBC cohorts, respectively, Additional File 2 – Tables 3 &4) nor in the whole MORGAM sample (Figure 4).

**Table 4:** *IL18RAP *gene haplotype frequencies in the MORGAM subcohorts

Polymorphisms										
					
rs11465670	rs4851581	rs1420106	rs1420105	rs11465673	rs11465702	FINRISK N = 700	ATBC N = 849	Sweden N = 136	PRIME/N.Ireland N = 143	PRIME/France N = 188
T	A	G	C	T	A	0.015	0.024	0.038	0.033	0.062

T	A	G	C	T	G	0.199	0.148	0.124	0.133	0.137

T	A	G	C	C	A	0.095	0.119	0.135	0.111	0.071

T	A	G	T	T	A	0.265	0.253	0.297	0.307	0.298

T	A	A	T	T	A	0.190	0.198	0.210	0.196	0.220

T	G	G	C	T	A	0.140	0.146	0.075	0.081	0.081

C	A	G	C	T	A	0.094	0.109	0.120	0.137	0.127

### Gene × Gene and Gene × environment analysis

**Figure 5:**
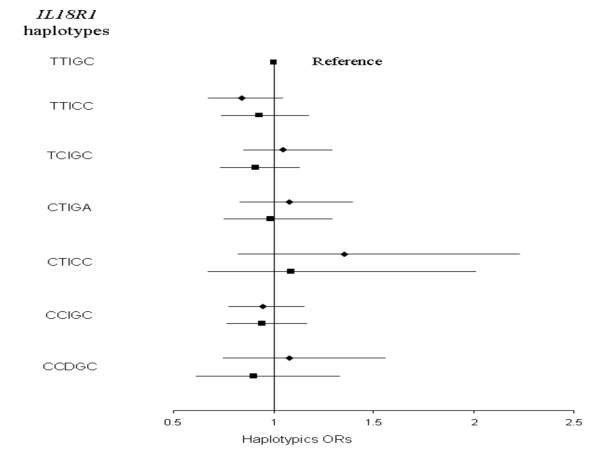
**Association between *IL18R1 *haplotypes and CVD risk in the whole MORGAM cohorts according to smoking status**. Haplotypic ORs [95% confidence interval] by comparison to the reference TTIGC haplotype were adjusted for cohorts, age, gender, separately in non-smokers (diamonds) and smokers (squares). The global tests of association were not significant (χ^2 ^= 4.796 with 6 df, p = 0.570 in non-smokers; χ^2 ^= 1.075 with 6 df, p = 0.983 in smokers). Polymorphisms are ordered according to their position on the genomic sequence.

**Figure 6:**
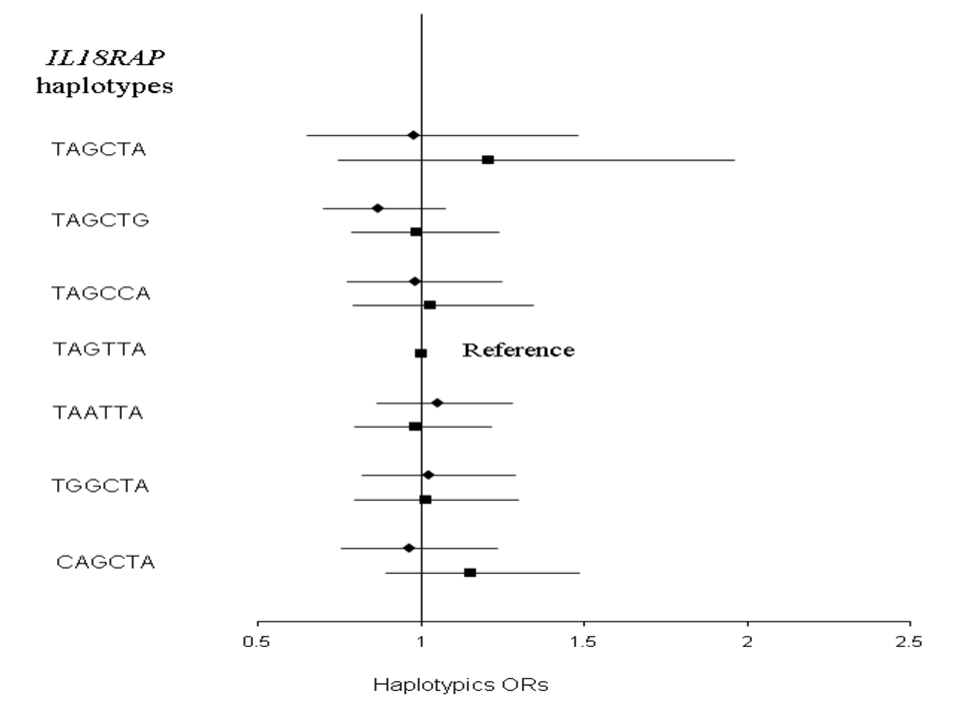
**Association between *IL18RAP *haplotypes and CVD risk in the whole MORGAM cohorts according to smoking status**. Haplotypic ORs [95% confidence interval] by comparison to the reference TAGTTA haplotype were adjusted for cohorts, age, gender, separately in non-smokers (diamonds) and smokers (squares). The global tests of association were not significant (χ^2 ^= 2.639 with 6 df, p = 0.853 in non-smokers; χ^2 ^= 2.028 with 6 df, p = 0.917 in smokers). Polymorphisms are ordered according to their position on the genomic sequence.

**Figure 7:**
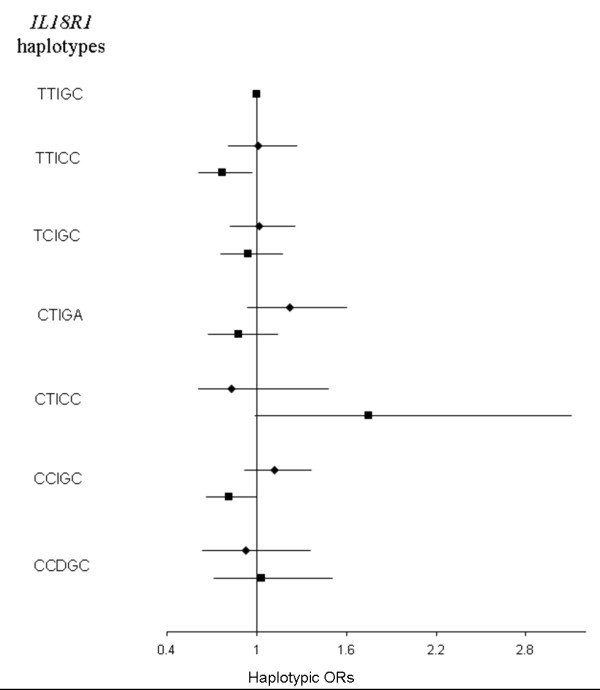
**Association between *IL18R1 *haplotypes and CVD risk in the whole MORGAM cohorts according to BMI**. Haplotypic ORs [95% confidence interval] by comparison to the reference TTIGC haplotype were adjusted for cohorts, age and gender, separately in subjects below (diamonds) and above (squares) the population-specific median of BMI. The global test of association was neither significant in the group of individuals below (χ^2 ^= 3.767 with 6 df, p = 0.708) nor above (χ^2 ^= 10.28 with 6 df, p = 0.113) the median of BMI. Polymorphisms are ordered according to their position on the genomic sequence.

**Figure 8:**
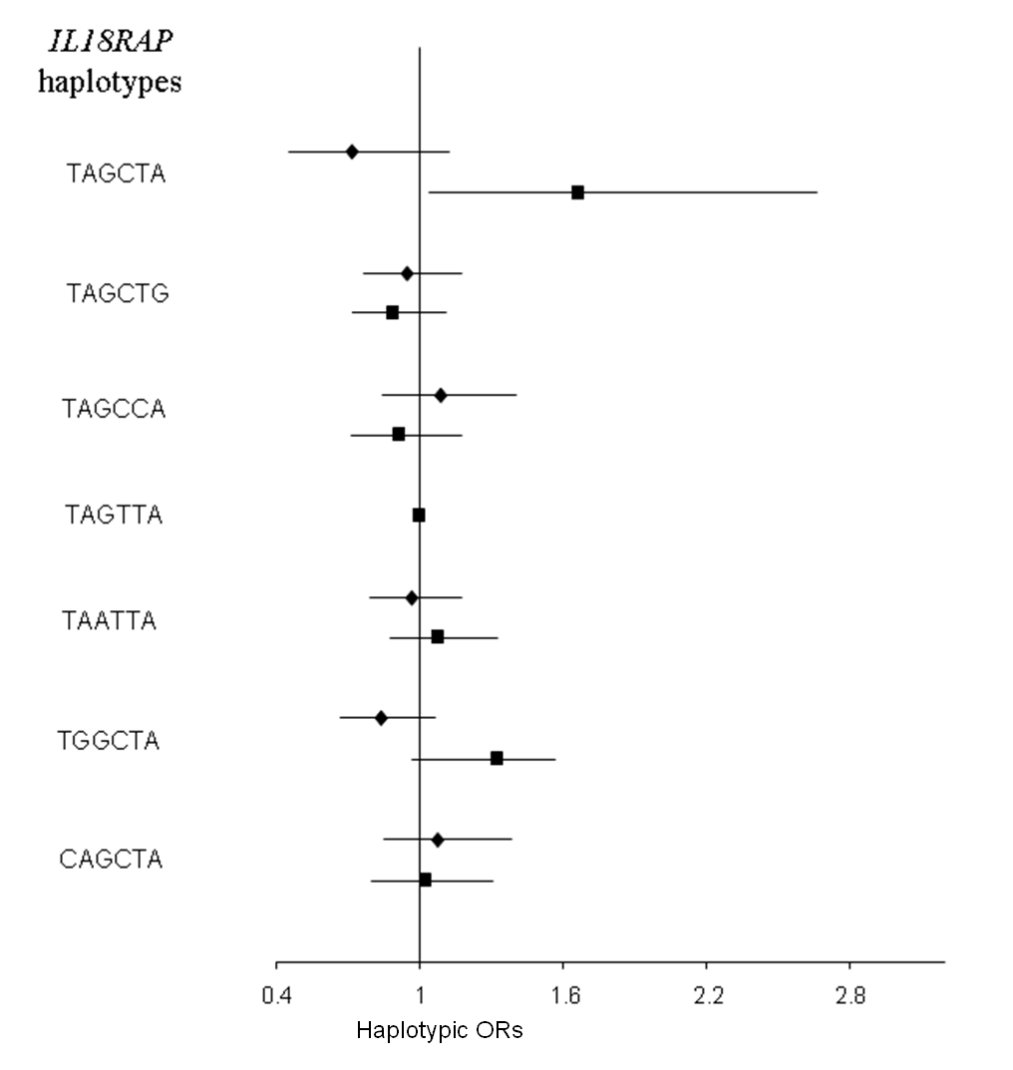
**Association between *IL18RAP *haplotypes and CVD risk in the whole MORGAM cohorts according to BMI**. Haplotypic ORs [95% confidence interval] by comparison to the reference TAGTTA haplotype were adjusted for cohorts, age and gender, separately for subjects below (diamonds) and above (squares) the population-specific median of BMI. The global test of association was neither significant in the group of individuals below (χ^2 ^= 5.379 with 6 df, p = 0.496) nor above (χ^2 ^= 11.18 with 6 df, p = 0.083) the median of BMI. Polymorphisms are ordered according to their position on the genomic sequence.

Finally, we were interested in testing whether *IL18R1 *and *IL18RAP *SNPs as well as 5 previously studied *IL18 *SNPs could interact with each other or with covariates including age, gender, smoking status and body mass index (BMI), to modify the risk of CVD. Except for the *IL18 *rs360717 allele whose effects have previously been found to be modulated by smoking [19], the search for interaction remained unfruitful. We did not detect any interaction between SNPs located in different genes nor between SNPs and environmental factors. For example, Figures 5 &6 illustrate the lack of differential effects of *IL18R1 *and *IL18RAP *haplotypes according to smoking while Figures 7 &8 display the absence of interaction with BMI.

## Discussion

*IL18R1 *and *IL18RAP *genes code for two receptors of the IL-18 cytokine that has been found associated with atherosclerosis and its cardiovascular complications[1]. As such, they are considered as good candidates for CVD. This motivated the present work in which *IL18R1 *and *IL18RAP *tag SNPs were assessed for association with CVD in different European cohorts as part of the MORGAM Project.

Eleven SNPs, 5 in *IL18R1 *and 6 in *IL18RAP*, were studied for association with CVD events in 5 European prospective cohorts, assembling more than 3000 subjects. Despite some minor differences in allele frequencies across cohorts, the haplotypic structures of these genes were broadly homogeneous. These allele frequencies were however comparable to those observed in HapMap Phase III European samples populations  or in NCBI dbSNP European database . None of the single SNPs, nor the inferred haplotypes, were found to be associated with CVD, results that are consistent with those reported in the AtheroGene study[9]. Because of the biological interaction between these two receptors and the IL-18 molecule, we also investigated whether SNPs of these 3 genes (*IL18R1*/*IL18RAP*/*IL18*) could interact with each other to modulate the risk of CVD. For this purpose, 5 previously studied *IL18 *SNPs were used [19]. No evidence for epistasis was observed in any of the 5 cohorts (data not shown). We had previously observed that one *IL18 *SNP interacts with smoking to modulate the risk of CVD [19]. However, no such interaction was detected with any of the *IL18R1 *and *IL18RAP *SNPs. Results shown in this report were obtained when CHD and stroke events were combined together but treating them separately did not provide further evidence of any specific genetic effects (data not shown).

It cannot be ruled out that the lack of association observed in this work could be due to sample size limitations. However, power calculations showed the MORGAM project to have a power of ~90% to detect the effect of a SNP with a minor allele frequency (MAF) of 0.10 and associated with an OR of at least 1.30. The same power would be achieved for an OR of 1.20 once the MAF was higher than 0.23. Additionally, our study is well powered to detect second-order interactions and has, for example, a power of ~70% to detect a SNP × smoking interaction where a SNP with MAF of 0.28 is associated with an OR of 1.30 in MORGAM smokers only [19]

## Conclusion

In conclusion, whereas *IL18R1 *and *IL18RAP *polymorphisms have been found associated with diseases such as schizophrenia, HSV1 seropositivity and atopic asthma [16,17], our analysis of *IL18R1 *and *IL18RAP *SNPs in 5 European prospective cohorts suggests that the variability of these genes are unlikely to contribute to modulate the risk of CVD in European populations.

## Competing interests

The authors declare that they have no competing interests.

*Financial competing interest*: none

## Authors' contributions

MLG carried out statistical analyses and prepared the initial manuscript draft. CP, MA and MD performed the genotyping. VS, FK, LT and DAT conceived the research plan and contributed to manuscript's writing. VN and DAT contributed to statistical data and data management analyses. KK, FC, PGW, JM, AE contributed to the conception, the design and the coordination of the study. VS, KK, FC, PGW, JM, FK, LT and AE performed critical review of multiple drafts of the manuscript. All authors read and approved the final manuscript

## Pre-publication history

The pre-publication history for this paper can be accessed here:



## Supplementary Material

Additional file 1 **Allele frequencies of the *IL18R1 *and *IL18RAP *SNPs in the MORGAM subcohorts**. Click here for file

Additional file 2 **Haplotype frequencies and odds ratios of IL18R1 and IL18RAP genes**. Table 1: Haplotype frequencies of the *IL18R1 *gene in the MORGAM cohorts (cases and non-cases). Table 2: Odds Ratio for CVD risk associated with *IL18R1 *gene haplotypes in the MORGAM cohorts. Table 3: Haplotype frequencies of the *IL18RAP *gene in the MORGAM cohorts (cases and non-cases). Table 4: Odds Ratio for CVD risk associated with *IL18RAP *gene haplotypes in the MORGAM cohorts. Click here for file
