# Supplementary material for: Lack of association between polymorphisms of the IL18R1 and IL18RAP genes and cardiovascular risk: the MORGAM Project
Source: BMC Med Genet. 2009 May 27;10:44. doi: 10.1186/1471-2350-10-44 (PMC2692850; doi:10.1186/1471-2350-10-44)
Supplement: Additional file 1 — Allele frequencies of the IL18R1 and IL18RAP SNPs in the MORGAM subcohorts. [file 1471-2350-10-44-S1.doc]

**Additional File 3 - Allele frequenciesa of the *IL18R1* and *IL18RAP* SNPs in the MORGAM subcohorts**

| Gene | SNP | FINRISK  N = 700 | ATBC  N = 849 | Sweden  N = 136 | PRIME/N.Ireland  N = 143 | PRIME/France  N = 188 | p-value^b^ |
| --- | --- | --- | --- | --- | --- | --- | --- |
| *IL18R1* | rs1420098 (T/C) | 0.39 | 0.40 | 0.43 | 0.41 | 0.37 | p = 0.160 |
|  | rs1420096 (T/C) | 0.46 | 0.46 | 0.49 | 0.48 | 0.54 | p = 2 10^-3^ |
|  | rs11465656 (I/D) | 0.04 | 0.04 | 0.03 | 0.05 | 0.04 | p = 0.278 |
|  | rs3732127 (G/C) | 0.19 | 0.17 | 0.15 | 0.18 | 0.19 | p = 0.019 |
|  | rs11465660 C/A) | 0.10 | 0.12 | 0.15 | 0.09 | 0.07 | p < 10^-4^ |
| *IL18RAP* | rs11465670 (T/C) | 0.09 | 0.11 | 0.13 | 0.15 | 0.12 | p < 10^-4^ |
|  | rs4851581 (A/G) | 0.15 | 0.15 | 0.08 | 0.07 | 0.09 | p < 10^-4^ |
|  | rs1420106 (G/A) | 0.19 | 0.19 | 0.21 | 0.20 | 0.26 | p = 2 10^-4^ |
|  | rs1420105 (C/T) | 0.47 | 0.46 | 0.49 | 0.48 | 0.53 | p = 3 10^-3^ |
|  | rs11465673 (T/C) | 0.10 | 0.12 | 0.13 | 0.11 | 0.07 | p = 0.012 |
|  | rs11465702 (A/G) | 0.18 | 0.15 | 0.13 | 0.15 | 0.14 | p = 2 10^-4^ |

^a^ Allele frequency of the minor allele

^b^ Test for difference of allele frequencies between subcohorts
