# Supplementary material for: Lack of association between polymorphisms of the IL18R1 and IL18RAP genes and cardiovascular risk: the MORGAM Project
Source: BMC Med Genet. 2009 May 27;10:44. doi: 10.1186/1471-2350-10-44 (PMC2692850; doi:10.1186/1471-2350-10-44)
Supplement: Additional file 2 — Haplotype frequencies and odds ratios of IL18R1 and IL18RAP genes. Table 1: Haplotype frequencies of the IL18R1 gene in the MORGAM cohorts (cases and non-cases). Table 2: Odds Ratio for CVD risk associated with IL18R1 gene haplotypes in the MORGAM cohorts. Table 3: Haplotype frequencies of the IL18RAP gene in the MORGAM cohorts (cases and non-cases). Table 4: Odds Ratio for CVD risk associated with IL18RAP gene haplotypes in the MORGAM cohorts. [file 1471-2350-10-44-S2.doc]

**Additional File 2 - Table 1 - Haplotype frequencies of the *IL18R1* gene in the MORGAM cohorts (cases and non-cases**)

| Polymorphisms | | | | | FINRISK | | ATBC | | Sweden | | PRIME/N.Ireland | | PRIME/France | |
| --- | --- | --- | --- | --- | --- | --- | --- | --- | --- | --- | --- | --- | --- | --- |
| rs1420098 | rs1420096 | rs11465656 | rs3732127 | rs11465660 | Non-cases N=609 | Cases  N=567 | Non-cases N=718 | Cases  N=552 | Non-cases  N=128 | Cases N=67 | Non-cases  N=135 | Cases  N=116 | Non-cases  N=182 | Cases  N=114 |
| T | T | I | G | C | 0.235 | 0.252 | 0.253 | 0.244 | 0.202 | 0.207 | 0.215 | 0.254 | 0.193 | 0.228 |
| T | T | I | G | A | 0.010 | 0.002 | 0.005 | 0.006 | 0.022 | 0.016 | 0.022 | 0.007 | 0.028 | 0.005 |
| T | T | I | C | C | 0.198 | 0.167 | 0.150 | 0.145 | 0.124 | 0.163 | 0.145 | 0.149 | 0.144 | 0.119 |
| T | C | I | G | C | 0.184 | 0.192 | 0.201 | 0.195 | 0.217 | 0.203 | 0.191 | 0.166 | 0.217 | 0.272 |
| C | T | I | G | A | 0.085 | 0.099 | 0.113 | 0.111 | 0.110 | 0.118 | 0.093 | 0.066 | 0.044 | 0.065 |
| C | T | I | C | C | 0.010 | 0.012 | 0.018 | 0.021 | 0.016 | 0.061 | 0.029 | 0.045 | 0.049 | 0.040 |
| C | C | I | G | C | 0.237 | 0.229 | 0.210 | 0.234 | 0.271 | 0.207 | 0.263 | 0.241 | 0.258 | 0.221 |
| C | C | D | G | C | 0.037 | 0.043 | 0.043 | 0.036 | 0.027 | 0.022 | 0.041 | 0.065 | 0.044 | 0.028 |
| Global Likelihood ratio test | | | | | χ^2^_(7)_ = 11.012  p = 0.138 | | χ^2^_(7)_ = 3.085  p = 0.877 | | χ^2^_(7)_ = 6.885  p = 0.441 | | χ^2^_(7)_ = 6.167  p = 0.520 | | χ^2^_(7)_ = 9.704  p = 0.206 | |

**Additional File 2 - Table 2 – Odds Ratio^*^ for CVD risk associated with *IL18R1* gene haplotypes** in the MORGAM cohorts

| Polymorphisms | | | | | FINRISK | ATBC | Sweden | PRIME/N.Ireland | PRIME/France |
| --- | --- | --- | --- | --- | --- | --- | --- | --- | --- |
| rs1420098 | rs1420096 | rs11465656 | rs3732127 | rs11465660 |
| T | T | I | G | C | reference | reference | reference | reference | reference |
| T | T | I | G | A | 0.201 [0.012 – 3.447] p = 0.268 | 1.241 [0.366 – 4.202] p = 0.729 | 0.704 [0.106 – 4.654] p = 0.715 | 0.204 [0.017 – 2.411] p = 0.207 | 0.190 [0.20 – 1.783] p = 0.146 |
| T | T | I | C | C | 0.791 [0.610 – 1.025] p = 0.076 | 0.994 [0.758 – 1.304] p = 0.968 | 1.379 [0.649 – 2.932] p = 0.403 | 0.893 [0.468 – 1.703] p = 0.732 | 0.763 [0.383 – 1.519] p = 0.441 |
| T | C | I | G | C | 0.967 [0.744 – 1.257] p = 0.803 | 1.002 [0.789 – 1.272] p = 0.986 | 0.940 [0.453 – 1.950] p = 0.868 | 0.756 [0.413 – 1.382] p = 0.363 | 1.062 [0.605 – 1.863] p = 0.834 |
| C | T | I | G | A | 1.111 [0.801 – 1.541] p = 0.527 | 1.014 [0.764 – 1.345] p = 0.924 | 1.059 [0.392 – 2.861] p = 0.910 | 0.581 [0.261 – 1.294] p = 0.184 | 1.234 [0.550 – 2.784] p = 0.606 |
| C | T | I | C | C | 1.098 [0.452 – 0.672] p = 0.836 | 1.204 [0.624 – 2.323] p = 0.580 | 4.999 [0.951 – 26.260] p = 0.057 | 1.075 [0.351 – 3.290] p = 0.899 | 0.617 [0.213 – 1.791] p = 0.375 |
| C | C | I | G | C | 0.935 [0.733 – 1.194] p = 0.592 | 1.150 [0.909 – 1.455] p = 0.243 | 0.777 [0.361 – 1.669] p = 0.517 | 0.721 [0.422 – 1.234] p = 0.233 | 0.669 [0.393 – 1.138] p = 0.138 |
| C | C | D | G | C | 1.140 [0.721 – 1.801] p = 0.575 | 0.870 [0.557 – 1.361] p = 0.543 | 0.860 [0.181 – 4.082] p = 0.849 | 1.316 [0.529 – 3.279] p = 0.555 | 0.424 [0.139 – 1.292] p = 0.131 |
| Global Likelihood ratio test | | | | | χ^2^_(7)_ = 11.012 p = 0.138 | χ^2^_(7)_ = 3.085 p = 0.877 | χ^2^_(7)_ = 6.885 p = 0.441 | χ^2^_(7)_ = 6.167 p = 0.520 | χ^2^_(7)_ = 9.704 p = 0.206 |

* Odds ratio [95% Confidence Interval] are given by comparison to the most frequent haplotype (reference). Odds ratio were adjusted on age, gender and smoking status under the assumption of haplotype additive effects.

**Additional File 2 - Table 3 - Haplotype frequencies of the *IL18RAP* gene in the MORGAM cohorts (cases and non-cases**)

| Polymorphisms | | | | | | FINRISK | | ATBC | | Sweden | | PRIME/N.Ireland | | PRIME/France | |
| --- | --- | --- | --- | --- | --- | --- | --- | --- | --- | --- | --- | --- | --- | --- | --- |
| rs11465670 | rs4851581 | rs1420106 | rs1420105 | rs11465673 | rs11465702 | Non-cases N=609 | Cases N=567 | Non-cases N=718 | Cases N=552 | Non-cases N=128 | Cases N=67 | Non-cases N=135 | Cases N=116 | Non-cases N=182 | Cases N=114 |
| T | A | G | C | T | A | 0.017 | 0.018 | 0.023 | 0.023 | 0.032 | 0.068 | 0.030 | 0.052 | 0.063 | 0.057 |
| T | A | G | C | T | G | 0.195 | 0.164 | 0.145 | 0.145 | 0.121 | 0.164 | 0.144 | 0.142 | 0.132 | 0.115 |
| T | A | G | C | C | A | 0.094 | 0.099 | 0.117 | 0.114 | 0.133 | 0.142 | 0.115 | 0.073 | 0.073 | 0.062 |
| T | A | G | T | T | A | 0.272 | 0.283 | 0.255 | 0.276 | 0.307 | 0.244 | 0.299 | 0.305 | 0.293 | 0.237 |
| T | A | A | T | T | A | 0.185 | 0.195 | 0.202 | 0.192 | 0.209 | 0.187 | 0.201 | 0.177 | 0.221 | 0.290 |
| T | G | G | C | T | A | 0.143 | 0.153 | 0.146 | 0.131 | 0.085 | 0.038 | 0.078 | 0.103 | 0.087 | 0.110 |
| C | A | G | C | T | A | 0.093 | 0.093 | 0.108 | 0.113 | 0.113 | 0.157 | 0.133 | 0.146 | 0.125 | 0.128 |
| Global Likelihood ratio test | | | | | | χ^2^_(6)_ =4.859  p = 0.562 | | χ^2^_(6)_ = 2.680  p = 0.848 | | χ^2^_(6)_ = 9.362  p = 0.154 | | χ^2^_(6)_ = 4.277  p = 0.639 | | χ^2^_(6)_ = 6.164  p = 0.405 | |

**Additional File 2 - Table 4 – Odds Ratio^*^ for CVD risk associated with *IL18RAP* gene haplotypes** in the MORGAM cohorts

| Polymorphisms | | | | | | FINRISK | ATBC | Sweden | PRIME/N.Ireland | PRIME/France |
| --- | --- | --- | --- | --- | --- | --- | --- | --- | --- | --- |
| rs11465670 | rs4851581 | rs1420106 | rs1420105 | rs11465673 | rs11465702 |
| T | A | G | C | T | A | 1.074 [0.542 – 2.130]  p = 0.837 | 0.914 [0.532 – 1.571]  p = 0.745 | 3.620 [1.116 – 11.744]  p = 0.032 | 1.527 [0.591 – 3.944]  p = 0.382 | 1.157 [0.531 – 2.519]  p = 0.714 |
| T | A | G | C | T | G | 0.797 [0.620 – 1.024]  p = 0.076 | 0.913 [0.701 – 1.187]  p = 0.496 | 1.829 [0.854 – 3.916]  p = 0.120 | 1.019 [0.567 – 1.829]  p = 0.951 | 1.256 [0.695 – 2.271]  p = 0.450 |
| T | A | G | C | C | A | 1.019 [0.744 – 1.396]  p = 0.906 | 0.896 [0.678 – 0.185]  p = 0.442 | 1.342 [0.595 – 3.025]  p = 0.478 | 0.641 [0.313 – 1.315]  p = 0.226 | 1.279 [0.641 – 2.556]  p = 0.485 |
| T | A | G | T | T | A | reference | reference | reference | reference | reference |
| T | A | A | T | T | A | 0.993 [0.774 – 1.273]  p = 0.955 | 0.871 [0.688 – 1.103]  p = 0.251 | 1.181 [0.605 – 2.304]  p = 0.626 | 0.904 [0.521 – 1.566]  p = 0.718 | 1.837 [1.073 – 3.143]  p = 0.026 |
| T | G | G | C | T | A | 1.045 [0.796 – 1.371]  p = 0.752 | 0.828 [0.638 – 1.075]  p = 0.157 | 0.483 [0.151 – 1.545]  p = 0.220 | 1.393 [0.688 – 2.822]  p = 0.357 | 1.586 [0.818 – 3.074]  p = 0.172 |
| C | A | G | C | T | A | 0.948 [0.688 - 1.306]  p = 0.744 | 0.962 [0.722 – 1.281]  p = 0.792 | 1.873 [0.860 – 4.076]  p = 0.114 | 1.071 [0.600 – 1.913]  p = 0.816 | 1.544 [0.856 – 2.784]  p = 0.149 |
| Global Likelihood ratio test | | | | | | χ^2^_(6)_ =4.859  p = 0.562 | χ^2^_(6)_ = 2.680  p = 0.848 | χ^2^_(6)_ = 9.362  p = 0.154 | χ^2^_(6)_ = 4.277  p = 0.639 | χ^2^_(6)_ = 6.164  p = 0.405 |

* Odds ratio [95% Confidence Interval] are given by comparison to the most frequent haplotype (reference). Odds ratio were adjusted on age, gender and smoking status under the assumption of haplotype additive effects.
